# Gene3D: expanding the utility of domain assignments

**DOI:** 10.1093/nar/gkv1231

**Published:** 2015-11-17

**Authors:** Su Datt Lam, Natalie L. Dawson, Sayoni Das, Ian Sillitoe, Paul Ashford, David Lee, Sonja Lehtinen, Christine A. Orengo, Jonathan G. Lees

**Affiliations:** 1 Institute of Structural and Molecular Biology, Division of Biosciences, University College London, Gower Street, London, WC1E 6BT, UK; 2 Department of Infectious Disease Epidemiology, Imperial College, St Mary's Campus, Norfolk Place, London W2 1PG, UK

## Abstract

Gene3D http://gene3d.biochem.ucl.ac.uk is a database of domain annotations of Ensembl and UniProtKB protein sequences. Domains are predicted using a library of profile HMMs representing 2737 CATH superfamilies. Gene3D has previously featured in the Database issue of NAR and here we report updates to the website and database. The current Gene3D (v14) release has expanded its domain assignments to ∼20 000 cellular genomes and over 43 million unique protein sequences, more than doubling the number of protein sequences since our last publication. Amongst other updates, we have improved our Functional Family annotation method. We have also improved the quality and coverage of our 3D homology modelling pipeline of predicted CATH domains. Additionally, the structural models have been expanded to include an extra model organism (*Drosophila melanogaster*). We also document a number of additional visualization tools in the Gene3D website.

## INTRODUCTION

Protein structural domains are compact structural modules within proteins and can be grouped into sometimes very large clusters of relatives showing clear evolutionary relatedness, termed homologous superfamilies. The CATH database takes structures from the Protein Data Bank (PDB), and from these identifies individual protein domains which are subsequently assigned to one of a few thousand homologous families (1). In the last decade the number of protein superfamilies in CATH has remained relatively stable, despite the ever increasing numbers of protein structures and protein sequences. Furthermore, it has long been known that domain superfamilies show an extremely skewed distribution in the numbers of protein sequences assigned to them (2). The largest domain superfamilies contain sequences that have greatly diverged in molecular function. Recently work has been carried out to help improve the functional purity of domain assignments by dividing the domain superfamilies into smaller functionally coherent groups termed Functional Families or FunFams (3). These FunFams greatly improve the ability to interpret the functions of an experimentally uncharacterized protein based on its domain assignments (3,4).

The Gene3D resource predicts domain superfamily assignments for tens of millions of protein sequences in UniProKB (5) and Ensembl (6,7) using HMMER3 sequence comparison tools (8) to match against the expertly-curated structural domains in CATH. In addition to domain superfamilies, Gene3D also provides the more functionally coherent FunFam assignments. The original FunFam assignment algorithm (named DFX) used in the previous release of Gene3D made use of experimentally-confirmed GO annotations to help guide the subdivision of domain superfamilies into FunFams. However since the last release an improved FunFam assignment algorithm, FunFHMMer, has been developed (8). Rather than solely relying on GO annotations, FunFHMMer uses specificity determining residues in multiple sequence alignments to subdivide domain superfamilies into FunFams. In the latest round of independent function prediction assessment (CAFA2), FunFHMMer-based methods showed significant improvements in function prediction performance with the method being ranked in the top five methods (out of 129) for functional annotation of query sequences. These improvements also tallied with our own benchmarks.

In summary, we have updated Gene3D domain assignments using the latest release of CATH (v4.1) and this has considerably expanded the numbers of protein sequences and genomes in the database. We have improved the functional annotations of these sequences using the FunFHMMer algorithm and have used the more accurate FunFams to improve our structural modelling pipeline. For the first time, we have incorporated Ensembl genomic coordinates into the database as a valuable aid to examine alternative splicing processes and we have incorporated knowledge of protein regions and mutations that affect protein interactions. We have also expanded our coverage of human mutations to the much larger set integrated by UniProt and included additional visualization tools in the website. Additionally, we have added in predictions of both disordered and foldable regions to help annotate protein regions currently missing domain assignments.

## New data in Gene3D

### Updated annotations and sequence set

The number of sequences in the database has increased by over 2-fold since our last release to 43 378 462 million sequences with over 19 471 cellular genomes now present. The number of CATH domain sequence assignments has increased from 26 to 54 million sequences belonging to 2737 CATH superfamilies.

The expansion in protein sequences in the database continues to be driven mainly by new bacterial genome sequences. Because of this continuing trend to more bacterial sequences it is essential to use a set of protein sequences common to both releases when comparing domain coverage between releases. For Ensembl sequences common to both releases (which predominantly consists of vertebrate genomes with a few non-vertebrate model organisms), there is a small but positive increase in domain coverage (i.e. the percentage of sequences with at least one domain annotated) from 70.8 to 71.4%. On a larger set of pan-taxonomic compara taxons from Ensembl Genomes (7), which includes many bacterial genomes, there is an increase in domain coverage from 61.1 to 61.5%. In terms of domain assignments in Gene3D there are a total of 48 891 183 domain assignments increasing to 65 792 139 merged CATH/Pfam domain assignments.

### New improved Functional Family assignments

In Gene3D v12, we introduced more specific domain assignments based on the functional sub-classification of CATH superfamily assignments (FunFams) (9). The new FunFam clusters in Gene3D v14 are generated using the FunFHMMer method (8), that analyses the combined multiple sequence alignment from two putative functional families to detect highly conserved positions and specificity-determining positions. Broadly speaking, the specificity determining residues are those identified as conserved amongst the sequences of one FunFam but not conserved or conserved differently in the other FunFam. For more details of this method we direct the reader to the FunFHMMer paper (8). The new FunFHMMer method generally produces smaller clusters than the previous method and identifies ∼110 000 functional families compared to ∼67 000 in the previous version of Gene3D. These new FunFam clusters are more functionally consistent with respect to the current GO annotation of their members than the previous method. Additionally, benchmarks have shown FunFHMMer to provide improved function prediction performance over the previous method. Additionally, preliminary results from the recent independent benchmark in CAFA2 showed FunFHMMer based predictions to be one of the very best methods and the top domain based annotation method. The preliminary results of CAFA-2 can be accessed from: https://github.com/idoerg/CAFA2-results.

### 3D structural models provided for selected organisms

As mentioned, FunFams are sub-classifications of CATH superfamilies derived using sequence signature analysis. Analysis of the FunFams produced by FunFHMMer shows that they represent structurally cohesive clusters well suited to be used for homology modelling (10). The structural coherence of these groups indicates that they have the potential to be used for the modelling of ‘Twilight zone’ proteins (proteins with pairwise sequence identity below 30% to their closest structural homologue). Our FunFam modelling pipeline assigns query domain sequences to FunFams following a scan against the in-house library of CATH-Gene3D FunFams. The query target sequence is assigned to the best matched FunFam, provided the E-value is less than 0.01. The template structure is selected as the sequence with known structure (within the same FunFam) that best matches the query sequence, following a BLAST scan of the query against the sequences of all possible target structures.

There is a manuscript in preparation describing the homology pipeline and its performance in more details. In brief, our analyses of the performance in using the new FunFams to build homology models for uncharacterized sequences show that they produce more good quality models (TM-score >0.50) than BLAST for close homologues (sequence identity ≥30%). TM-scores have previously been shown to be useful in assessing model quality (11). Although the FunFam modelling pipeline generates fewer remote homologues models compared to PSI-BLAST (12) and HHsearch (13), it provides the highest percentage of good quality models. Furthermore, the FunFam modelling pipeline produces more good quality models for remote homologues, than HHsearch, whether they are using the same template or different templates, than those selected by PSI-BLAST or HHsearch. Our analyses suggest that by using the FunFam modelling pipeline, we improve the alignment of the query sequence against the relative providing the template structure. This in turn improves the homology modelling of the query.

Our FunFam modelling pipeline has been applied to model domain sequences in Human and *Drosophila melanogaster* genomes. The FunFam modelling pipeline generates 24 and 10% more human models compared to the classical BLAST and PSI-BLAST approaches, respectively (Supplementary Figure S1). In cases where FunFams cannot obtain a good template match, HHsearch is used to obtain extra coverage. Compared to the previous release, we have added in structural models for the *D.melanogaster* genome and we are in the process of adding structural models for an additional eight model organisms. These are expected to be completed by the time this manuscript is published.

### Other database additions

As another new feature in this release of the Gene3D database, we now integrate the full set of mutations stored in UniProtKB. This includes both manually curated mutations and those automatically imported from the Ensembl genomes project (5).

Alternative splicing is an important process for expanding the functional repertoire of complex organisms. We have imported genomic coordinate data for Ensembl genomes, which allows protein features such as domains to be mapped back to their parent exons.

For binary protein interactions we have extracted information on sub-regions of a protein sequence where there is some evidence in IntAct (14), that this region has a role in mediating the protein interaction or affecting the strength of the interaction. This type of data helps to determine which domains, if any, are most likely contribute to an interaction and also, through structural modelling, how mutations in the sub-domain regions might alter a protein interaction.

## WEBSITE UPDATES

The website has been augmented with additional datasets and visualization tools, for brief descriptions of these additions see below.

### Protein page updates

**Figure 1.**
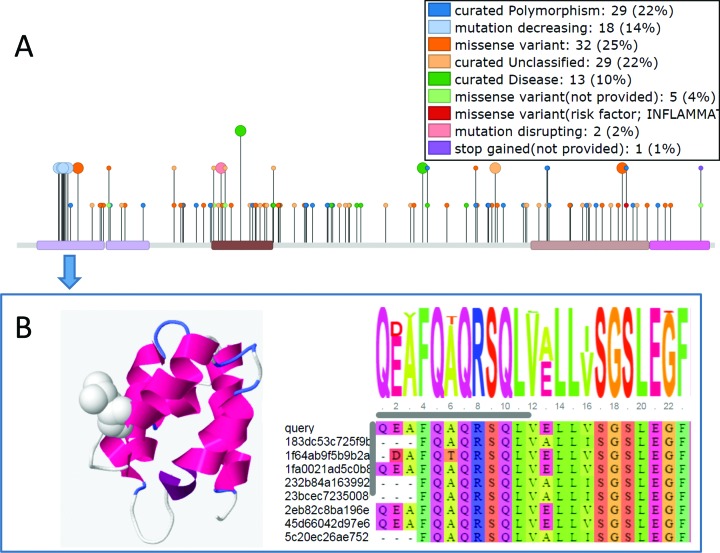
(**A**) mutation data displayed for NOD2_HUMAN and summarized for each domain (**B**) Clicking on a domain provides a link to the domain page where structural models and sequence alignments of the domain are available. Various data including which mutations affect interactions are displayed and clicking on one of these mutations shows the residue in ‘space-fill’. In this case we highlight a mutation in the N-terminal domain of NOD2 which decreases its protein interaction strength with ATG16L1. The alignment is built from the FunFam seed alignment and the query domain is added using the MAFFT ‘add_sequences’ function.

A large amount of mutation data has become available in recent years. It has been shown that domain-based analysis of mutation data can help when interpreting how a mutation contributes to disease (15). To help with the visualization of the mutation data, superposed on the Gene3D domains we have added in a needle plot graphic implemented using the muts-needle-plot package (16) (Figure 1A). The mutation data include SNPs and disease mutations collated by UniProt and point mutations affecting protein interactions curated by IntAct. The graphic includes a legend showing a summary of the numbers and types of mutations. Clicking on a domain in the muts-needle-plot visualization tool provides a quick overview of the mutations for that domain. Additionally, clicking on a domain produces a link to go through to the domain sequence and structure view, where details of the mutation on the domain can be investigated in more detail (Figure 1B).

Disordered regions predicted by IUPRED-long (17) are now displayed along with the domains. This is particularly useful for domain sized regions of a protein with no domain assignments. Regions that are predicted as disordered are less likely to fold into globular domains than non-disordered regions. However, it is important to note that disordered regions can retain some intrinsic order and folding can often arise when a disordered region binds with another protein. As a further complement to this we provide IUPRED-glob predictions. IUPRED-glob predicts globular domains. As it does not depend on homology-based methods, IUPRED-glob is able to predict ‘orphan’ domains. IUPRED-glob thus complements intrinsic protein disorder and homology-based domain predictions from Gene3D. An example of where a domain assignment may have been missed is for TAF14_YEAST, which appears to have a C-terminal foldable region with no predicted CATH or Pfam domain (18).

### Domain page updates

When the sequence can be mapped to a FunFam we provide a multiple sequence alignment of the query domain sequence aligned to the other FunFam members. We have updated the display of this alignment to use the BioJS MSA viewer (http://msa.biojs.net/) which allows for much improved visualization options and can cope with large alignments (Figure 1B).

A model is deemed reliable enough to display if they have either a GA341 score > = 0.7 or a normalized DOPE score <0 (as described in (19)). Additional Ramachandran plots are displayed to allow for further quality checks.

If a structural model is available, clicking on a residue in the top bar of the MSA viewer highlights the selected residue on the structural model. It is possible to highlight many other features stored in Gene3D onto the structure. For example, we have added the ability to display the position specific data in UniProt which contains over 20 different feature types, such as active-sites. Additionally, any regions annotated with information on their role in mediating proteins interaction can be displayed using data imported from IntAct. The IntAct imported data also contains information on mutations which can alter the strength of the interaction (Figure 1B).

For a modelled structure it is possible to highlight on the structure which exons code for different stretches of protein sequence. Clicking on an exon highlights that section on the structure whilst clicking the ‘colour by exons’ alternates the colour of consecutive exons in the structure. CRISPR-CAS9 developments (20) are making manipulation of coding sequences much easier and tools for mapping genomic coordinates to protein structure/function will be useful for helping in experimental design. In the exon table there is information on the inclusion level of the exon in all transcripts of the gene.

### Domain family page updates

**Figure 2.**
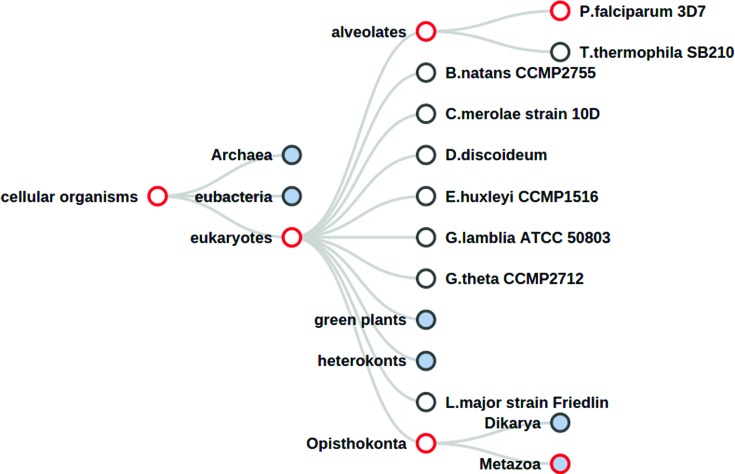
Domain family tree view of the domain superfamily 2.10.10.10 in the pan-Ensembl (compara) taxonomic tree. A red border indicates that taxonomic level has at least one gene assigned with the query domain family. Nodes filled with a blue colour indicate the child taxonomic levels are hidden, and clicking on these nodes will expand to show extra species.

We have improved our genomic comparison tools making using of D3.js (21) based tree display tools. In addition, subsets of Ensembl can now be visualized separately (pan-Ensembl, Ensembl Metazoa, Ensembl Protists, etc.). The tree can be zoomed and the nodes collapsed to save space. Moving the mouse cursor over a node gives further details on taxon information, domain counts, etc. The presence or absence of a domain family in a specific part of the tree can be identified by means of the colour of the nodes border. As an example we can see that in the pan-compara tree distribution for the 2.10.10.10 superfamily (fibronectin-type II domains) (Figure 2), the family is found predominantly in Metazoan species.

### Domain-protein interactions page

There are many high quality protein interaction resources available with advanced network display tools (14,22). It is known that protein interactions are frequently mediated by domains. In some cases, such as for SH3 and SH2 domains, mediating protein interactions is the main function of the domain. For a number of binary protein interactions there is information on sub-regions of the proteins that participate or influence the interaction. Many of these sub-regions overlap with domain regions in Gene3D. To bring all the protein interactions together for a particular protein and frame it in the context of domains we have built a visualization tool that assimilates the domain annotation data with the sub-region protein interaction data. Given a search with a query protein, if this information is present in Gene3D, then a link is provided to this network visualization page. Some examples of proteins with this type of sub-region interaction annotation can be found on the Gene3D examples page (http://gene3d.biochem.ucl.ac.uk/examples).

In the interaction networks the query proteins domains are displayed as circular nodes. Inside the nodes we show an image of a representative structure from the domain superfamily to which the domain belongs. A link between a domain node and a protein node indicates there is experimental annotation indicating that a sub-region of the protein, which overlaps with the domain, affects the interaction with that protein. The links between domains and proteins become thicker as a greater proportion of the annotated sub-region is covered by the domain. A red link indicates terms such as ‘sufficient to bind’ etc. and clicking on an edge joining a protein and domain gives further details along with links back to the source resource that provided the annotation. A blue link indicates instances where a mutation is known to affect the interaction. A sliding bar allows the user to filter out those links between domains and proteins where the sub-region interaction annotation has a relatively small overlap with the domain.

**Figure 3.**
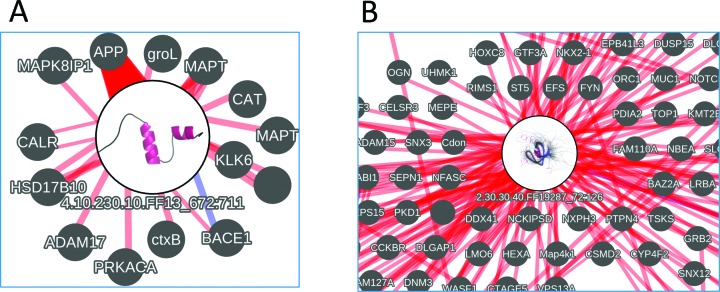
Example highly connected domains from the Domain-protein interaction network view for (**A**) A4_HUMAN. The large circular node (with a representative domain superfamily image inside) shows the domain with most overlapping interaction annotations (from the A4_HUMAN protein) and the label shows its superfamily code and region on the full protein sequence. The small grey nodes show proteins that this domain of A4_HUMAN is likely to mediate interactions with (NB. In this image the interactions are filtered to only include those interactions where greater than 50% of the sub-region annotation is covered by the domain). The width of the edge indicates the proportion of the sub-region annotation that is covered by the domain. Blue links indicate cases where a mutated residue in the domain has been shown to affect the interaction. (**B**) Interactions for ABL1_HUMAN zoomed in on the SH3 Domain of ABL1_HUMAN. The Networks are built using cytoscape.js.

As an example, for the amyloid protein A4_HUMAN ,there is evidence for its C-terminal domain having a role in mediating interactions with other proteins (Figure 3A). As another example, for the human ABL1 protein we can see that one of its domains is involved in a particularly large number of interactions (Figure 3B). If the domain has a modelled structure in Gene3D it is possible to click through to the model and inspect where the interaction features are on the structure.

### Alternative splicing annotation page

Clicking on the ‘Splicing Browser’ link in the navigation bar at the top of the page produces a table of entries where there is some isoform specific functional annotation from UniProt. Entering text in the search box of this section filters the table and highlights the matched text. For example, entering search terms ‘location’ and ‘nucleus’ shows isoform annotations where there is information that this isoform may have a role in nuclear localization.

## DATA DOWNLOADS

We provide the same downloads as for previous releases at the usual ftp site (ftp://ftp.biochem.ucl.ac.uk/pub/gene3d_data/CURRENT_RELEASE/). We have added domain annotations for many key organisms in the ‘TAXONS’ sub-directory of the FTP site.

## CONCLUSION

The sequence datasets continue to grow yet the number of domain families remains rather constant at 2737. Gene3D provides large scale annotation of these domain superfamilies for over 40 million proteins. Concurrently, domain assignments are becoming more useful through developments in domain based function/structure assignment and integration of other sequence specific (interaction, function, mutation) data. As the datasets grow, improvements in visualization tools are needed to make best use of the data. It is notable that we make use of several BioJS (23) components in this work highlighting the utility of such projects.
